# Antibacterial Activity and Mechanism of Ginger Essential Oil against *Escherichia coli* and *Staphylococcus aureus*

**DOI:** 10.3390/molecules25173955

**Published:** 2020-08-30

**Authors:** Xin Wang, Yi Shen, Kiran Thakur, Jinzhi Han, Jian-Guo Zhang, Fei Hu, Zhao-Jun Wei

**Affiliations:** 1 School of Food and Biological Engineering, Hefei University of Technology, Hefei 230601, China; 18158951863@163.com (X.W.); 2019111359@mail.hfut.edu.cn (Y.S.); kumarikiran@hfut.edu.cn (K.T.); zhangjianguo@hfut.edu.cn (J.-G.Z.); 2 College of Biological Science and Technology, Fuzhou University, Fuzhou 350108, China; hjz419@fzu.edu.cn; 3 School of Biological Science and Engineering, North Minzu University, Yinchuan 750021, China

**Keywords:** ginger essential oil, chemical composition, antibacterial mechanism, bacterial cell membrane, bacteria lysis-related gene

## Abstract

Though essential oils exhibit antibacterial activity against food pathogens, their underlying mechanism is understudied. We extracted ginger essential oil (GEO) using supercritical CO_2_ and steam distillation. A chemical composition comparison by GC-MS showed that the main components of the extracted GEOs were zingiberene and α-curcumene. Their antibacterial activity and associated mechanism against *Staphylococcus aureus* and *Escherichia coli* were investigated. The diameter of inhibition zone (DIZ) of GEO against *S. aureus* was 17.1 mm, with a minimum inhibition concentration (MIC) of 1.0 mg/mL, and minimum bactericide concentration (MBC) of 2.0 mg/mL. For *E. coli,* the DIZ was 12.3 mm with MIC and MBC values of 2.0 mg/mL and 4.0 mg/mL, respectively. The SDS-PAGE analysis revealed that some of the electrophoretic bacterial cell proteins bands disappeared with the increase in GEO concentration. Consequently, the nucleic acids content of bacterial suspension was raised significantly and the metabolic activity of bacteria was markedly decreased. GEO could thus inhibit the expression of some genes linked to bacterial energy metabolism, tricarboxylic acid cycle, cell membrane-related proteins, and DNA metabolism. Our findings speculate the bactericidal effects of GEO primarily through disruption of the bacterial cell membrane indicating its suitability in food perseveration.

## 1. Introduction

Food-borne diseases are considered an extremely important public health concern posing worldwide risks to the food industry and consumers, mainly due to food contamination caused by two key pathogens *Escherichia coli* and *Staphylococcus aureus* [1,2]. Among the various intrinsic determinant factors for food deterioration, the presence of certain pathogenic microorganisms (bacteria, fungi, and virus) can cause spoilage and decay which impede to socio-economic development. Under certain conditions, *E. coli* can cause gastrointestinal tract infections, urinary tract infections and other local tissue and organ infections [3]; Meanwhile, *S. aureus* as an opportunistic pathogen that can produce enterotoxins that lead to food poisoning [4]. Therefore, the quest for potential antibacterial agents with precise mechanisms of action has become an essential drive among food scientists.

With the continuous improvement of living standards, consumers have expressed higher requirements for preservatives in food additives, expecting not only to have obvious preservative effects, but also to ensure the safety and non-toxicity [5]. However, a large number of studies have proved that synthetic preservatives are often endowed with considerable toxicity and may even lead to deformity and cancer development [6]. In the recent years, the main research focus is to include spice extracts and natural essential oils in food preservation regimes targeting extended shelf life and the safety of food commodities [7]. Therefore, it is of interest to find novel natural antibacterial agents to improve the shelf life of food products and protect them from pathogenic spoilage.

Essential oils (EOs) are volatile aromatic substances extracted from different parts of plants. Until today, the main conventional methods used for EO extraction and separation include steam distillation and supercritical CO_2_ extraction [8,9]. Because of the appreciable antibacterial properties, different kinds of EOs have been widely used as excellent substitutes for chemical-based preservatives in recent years [10]. Previous studies have revealed the antibacterial action of EOs against *Listeria monocytogenes* [11], *S. albus*, *Bacillus subtilis, Salmonella typhimurium, Shigella dysenteriae,* and *E. coli* [12]. Among the natural plants, ginger is a kind of perennial herb belonging to the Zingiberaceae family. It originated from in the tropical regions of south east Asia and since then is widely cultivated across China [13]. Ginger is used as a spice in foods and beverages because of its characteristic spicy aroma and taste. In addition, it is an excellent source of many bioactive compounds, including bioactive phenols (gingerols, shogaols, and zingerones) [14]. Ginger essential oil (GEO) is the volatile oil extracted from the root of ginger. Due to its unique fragrance and biological activity, it has a very broad development prospects in the pharmaceutical, food, and cosmetics industries [15]. In particular, GEO is generally considered a safe natural compound, which has its application value in the treatment of gastrointestinal and respiratory diseases [16].

GEOs have been extensively investigated with a special focus on their antioxidant, antifungal, and antibacterial activities, as well as their ever-increasing applications in food preservation [17]. Nanocapsules based on GEO showed a remarkable antibacterial activity against *E. coli*, *Bacillus subtilis,* and *S. aureus* [18]. A nanoemulsion-based edible sodium caseinate coating containing GEO was applied to chicken breast fillets to extend their shelf life [19]. Another study revealed that GEO application could prolong the life of the leaves and improved the visual quality after harvest [20]. However, few studies have reported the effects of GEOs on bacteria-related gene expression profile responsible for the resulting antibacterial mechanism.

Although the chemical composition and antimicrobial attributes of EOs have been studied previously, the comparison of their chemical constituents using different extraction methods as well as the linked primary antibacterial mechanisms still remain unexplored. Therefore, the purpose of this study was to extend the functional potential of GEOs by investigating their antibacterial activity against two major food-borne bacteria and also to examine the possible underlying antibacterial mechanisms. Meanwhile, the effect of GEO on the mRNA expression of related genes in the two bacteria were evaluated, which is the key finding of the current study. Our study provides a basic understanding of the antibacterial mechanism of GEO and supports its applications for the industrial preservation purposes.

## 2. Results

### 2.1. Chemical Component Analysis of GEO

The chemical composition of the GEOs is shown in Table 1, which reveals 20 kind of active components, accounting for 84.727% and 78.325% of the total content. The volatile oils of ginger obtained by the two extraction methods included characteristic volatile components such as zingiberene, α-curcumene, and 6-gingerol (Table 1). The highest content among all the ingredients corresponded to zingiberene, followed by α-curcumene. After testing, the chemical components in the GEO extracted by the two methods were found to be similar, but to differ in the amounts. In addition, compared with steam distillation, more monoterpenes with shorter retention times such as α-pinene, camphene, and sabinene were detected in GEO extracted by the supercritical CO_2_ method. This might be due to the fact that when GEO is extracted by steam distillation, it is usually performed at a higher temperature (100 °C) and the extraction equipment is relatively open, which results in the loss of thermally sensitive and active components from the volatile ginger oil. Thus, the GEO obtained through the supercritical CO_2_ extraction was selected as the raw material for subsequent experiments.

### 2.2. Antibacterial Activity

#### 2.2.1. Determination of DIZ, MIC, and MBC in GEO against Test Microorganisms

**Figure 1:**
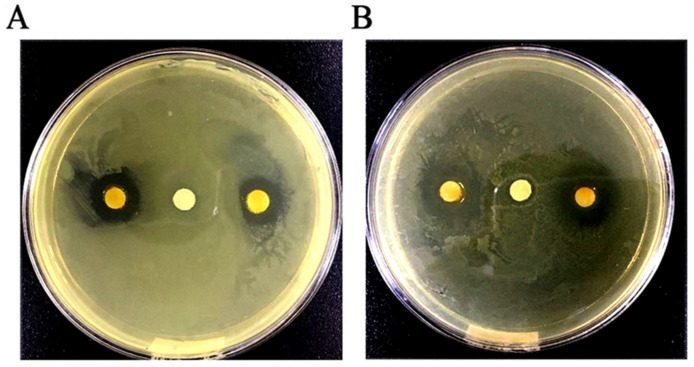
Determination of diameter of inhibition zone in GEO (16 mg/mL) treated *E. coli* (**A**) and *S. aureus* (**B**). In (**A**), the left and right corner represent GEO treated *E. coli.* In (**B**), the left and right corner represent GEO treated *S. aureus.* In (**A**) and (**B**), the middle spot represents the control group without GEO.

As shown in Figure 1, the antibacterial activity of GEO was qualitatively evaluated by measuring the size of the DIZ. The results showed that the means of DIZ for *E. coli* and *S. aureus* were 12.3 mm and 17.1 mm, respectively. As shown in Table 2, the antibacterial effect of GEO was quantitatively analyzed by the values of MIC and MBC against *E. coli* and *S. aureus*, where the MIC values were 2.0 mg/mL and 1.0 mg/mL; and the MBC values were 4.0 mg/mL and 2.0 mg/mL, respectively.

#### 2.2.2. Bacterial Growth Curve Analysis

**Figure 2:**
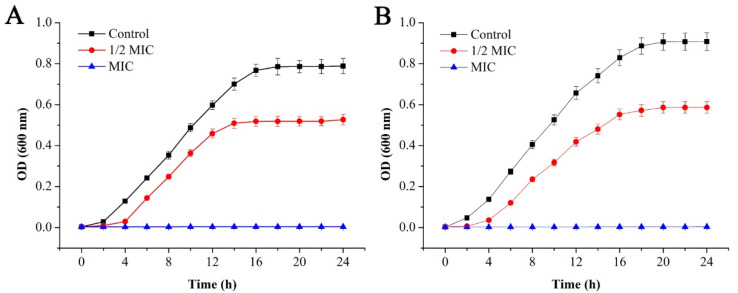
Effect of GEO on the growth of *E. coli* (**A**) and *S. aureus* (**B**).

To further verify the antibacterial effect of GEO on *E. coli* and *S. aureus*, the effects of GEO on generation time (Table 3) and the specific growth rate (Table 4) of *E. coli* and *S. aureus* were investigated, and the growth curve analysis of *E. coli* and *S. aureus* under different concentrations of GEO is shown in Figure 2.

Compared with the treatment group, there was a rapid rise in the optical density values of the control group, while the optical density of *E. coli* remained unchanged after about 16 h, and it remained constant for *S. aureus* for about 20 h, which was mainly related to the growth cycle of the bacteria. When the concentration of GEO approached 1/2 MIC level, bacterial growth was delayed with an extended lag time, and the final optical density values decreased significantly (*P* < 0.05) compared with the control group. Among both bacteria, the decline was more prominent in *S. aureus* than *E. coli*, indicating the inhibited growth pattern of both bacteria to some extent. When the concentration of GEO reached the MIC level, the optical density value remained basically unchanged. For *E. coli* and *S. aureus*, their generation time decreased significantly (*P* < 0.05) with the increase of GEO concentration (Table 3). At the same time, their generation time was dependent on their culture time (2 h to 24 h). In addition, their specific growth rate decreased significantly (*P* < 0.05) with the increase in culture time after treatment with GEO (Table 4).

### 2.3. Antibacterial Mechanism

#### 2.3.1. Membrane Potential 

**Figure 3:**
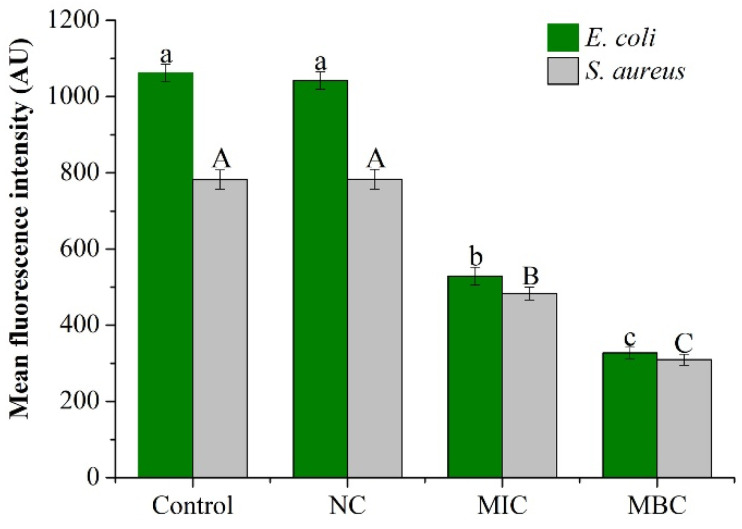
Effect of different concentrations of GEO on MP of *E. coli* and *S. aureus.* The *P* < 0.05 represents the significant differences which are depicted in the form of alphabets (the capital and small letters represent the different organisms and different superscripts represent significant differences.).

Changes in bacterial membrane potential affected the bacterial metabolic activity as shown in Figure 3. For the two bacteria, compared with the control group and the negative control group, the mean fluorescence intensity of the MIC and MBC levels of the EOs treatment group showed a significant downward trend (*P* < 0.05). No significant difference was observed between the control group and the negative control (P > 0.05). When the concentration of GEO approached the MIC level, the mean fluorescence intensities of *E. coli* and *S. aureus* were decreased by 50.17% and 38.27%, respectively. At the same time, under the treatment of MBC level, mean fluorescence intensities of *E. coli* and *S. aureus* were decreased by 69.18% and 60.48%, respectively.

#### 2.3.2. SDS-PAGE Electrophoresis of the Bacterial Proteins

**Figure 4:**
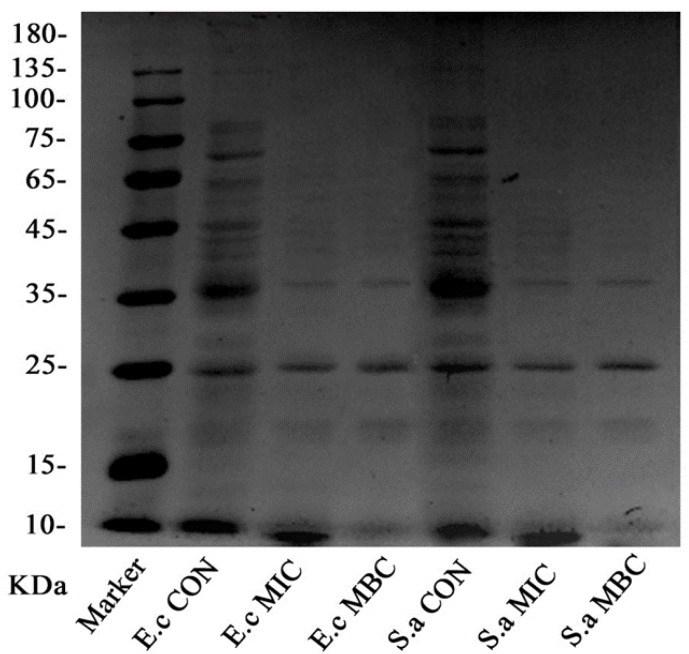
The gel electrophoresis picture of *E. coli* and *S. aureus* intracellular proteins.

The experimental results of SDS-PAGE are shown in Figure 4. For both the organisms, the bacteria in the control group showed clearer and more abundant protein bands, while the protein bands in the MIC group and MBC group were faded, and some of the protein bands even disappeared. 

Particularly, compared with the control group, the bands above 35 kDa in the EO treatment group disappeared, leaving behind bands with small molecular weights. Under MIC concentration treatment, the bands which still remained intact were 35 kDa, 25 kDa, and 10 kDa; while in case of the MBC concentration treatment, even the 10 kDa molecular weight bands vanished. 

#### 2.3.3. Determination of Bacterial Cell Proteins Concentration

**Figure 5:**
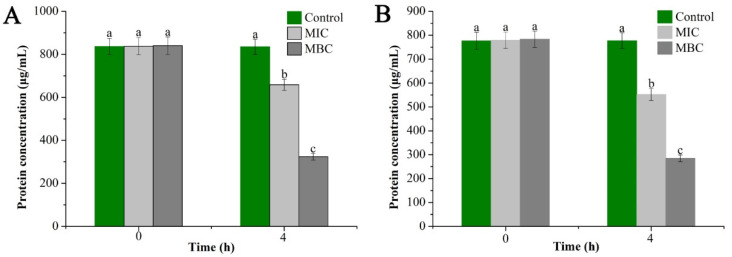
Effect of GEO on intracellular proteins content of *E. coil* (**A**) and *S. aureus* (**B**). The *P* < 0.05 represents the significant differences which are depicted in the form of alphabets (a, b, and c).

To further extend the above results, the bacterial proteins before and after treatment with the EO were extracted and their contents were measured as shown in Figure 5. The measurement results showed that the proteins in the bacterial cells were decreased (significant downward trend) with the increase in GEO concentration. Specifically, *E. coli* cell protein content was decreased from 836.29 µg/mL in the control group to 324.02 µg/mL in the MBC group, while *S. aureus* showed a decrease from 776.87 µg/mL in the control group to 285.1 µg/mL in the MBC group. Meanwhile, there were significant differences in bacterial protein content after 4 h of treatment with different concentrations of GEO (*P* < 0.05), which was consistent with the results obtained from SDS-PAGE analysis. 

#### 2.3.4. Effect of GEO on cell Membrane Integrity

**Figure 6:**
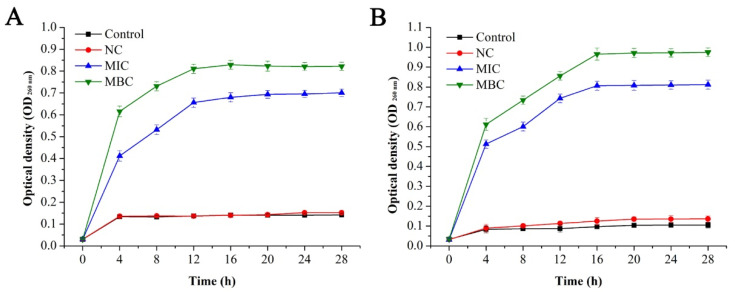
Effect of GEO on cell membrane integrity of *E. coli* (**A**) and *S. aureus* (**B**).

As shown in Figure 6, the nucleic acid content (OD_260nm_) of the blank group and the negative control group did not change significantly with time (*P* > 0.05), while, the nucleic acid content of the bacterial suspension of the EO treatment group increased significantly (*P* < 0.05). The absorbance of nucleic acid content increased from 0.0303 to 0.7009 and 0.822 at the MIC and MBC level at 16 h, respectively and then after, remained basically unchanged. The absorbance of nucleic acid content of *S. aureus* increased from 0.0312 to 0.812 and 0.975 at the levels of MIC and MBC at 20 h, respectively, and remained stable afterwards.

#### 2.3.5. Effect of GEO on the Bacteria Lysis-Related Gene Expression

**Figure 7:**
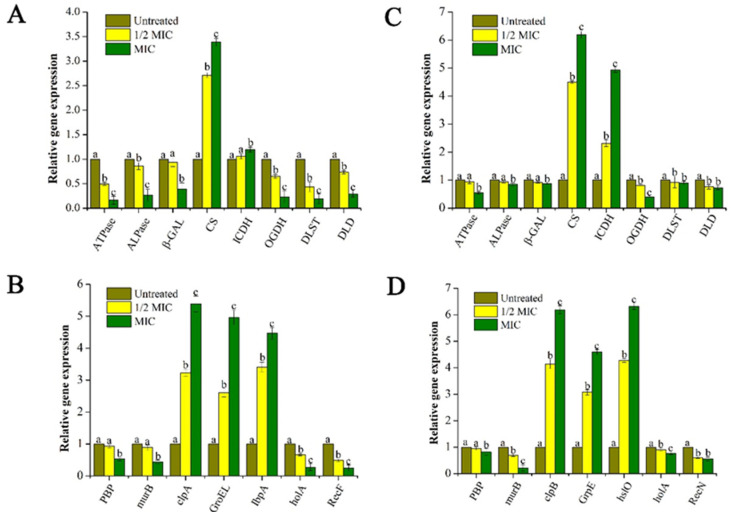
Effect of different concentrations GEO on expression of Lysis-Related Genes in *E. coli* (**A**,**B**) and S. aureus (**C**,**D**). The *P* < 0.05 represents the significant differences which are depicted in the form of alphabets (a, b, and c).

After being treated with GEO for 30 min, the expression levels of some selected genes were analyzed for the two bacteria and the results are shown in Figure 7. Compared with the internal control gene, among the 15 representative genes, five genes were up-regulated and 10 genes were down-regulated. Specifically, some genes related to the bacterial energy metabolism, including adenosine triphosphatase (ATPase), alkaline phosphatase (ALPase), and β-galactosidase (β-GAL) were significantly (*P* < 0.05) down-regulated. At the same time, with the increased concentration of GEO, the down-regulation trend became more pronounced (*P* < 0.05).

In addition, the expression of some key genes in the tricarboxylic acid (TCA) cycle pathway was analyzed, and it was found that some upstream genes in the TCA cycle including citrate synthase (CS) and isocitrate dehydrogenase (ICDH) were significantly up-regulated (*P* < 0.05), while downstream genes including oxoglutarate dehydrogenase (OGDH), Dihydrolipoamide succinyltransferase (DLST), and dihydrolipoamide dehydrogenase (DLD) showed a downregulation trend, which may be attributed to inhibited TCA cycle after GEO treatment resulting in the accumulation of upstream genes, while downstream genes were not expressed at all. 

**Figure 8:**
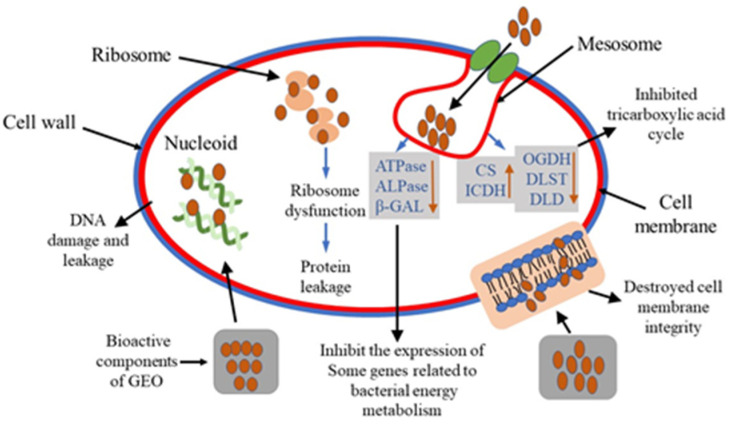
Possible antibacterial mechanism of action of GEO.

Furthermore, penicillin-binding protein (PBP) and acetylenolpyruvoylglucosamine reductase (murB) are a type of genes encoding cell membrane-related proteins. In our study, the down-regulation of these genes suggested that cell membranes were damaged after GEO treatment. And some genes including ATP-dependent Clp protease (clpA, clpB), Heat shock protein (GroE, GrpE), small heat shock protein (IbpA, hslO) were up-regulated significantly (*P* < 0.05). Meanwhile, genes related to DNA metabolism were down-regulated, including DNA repair proteins (RecF, RecN) and DNA polymerase (holA). Furthermore, compared to control and 1/2 MIC, MIC group showed significant increase for CS, clpA, GroEL, IbpA in *E. coli* and CS, ICDH, clpB, GrpE, and hslO for *S. aureus*. For these genes, the superscript “c” represented the MIC group with the highest fold increase compared to the control and 1/2MIC groups. The possible mechanism of GEO inhibiting bacterial growth is vividly demonstrated in Figure 8.

## 3. Discussion

It is well-known that GEOs are complex mixtures of volatile compounds produced as secondary metabolites in ginger, widely used as potential alternatives for chemically synthesized antimicrobials and antioxidants [20]. Mesomo et al. [15] reported that the sesquiterpenoids and monoterpenoids found as major compounds of GEOs were phenolic in essence. As expected, this is consistent with our GC-MS component analysis results. In another study, EOs were obtained from *Zingiber zerumbet* L powders by hydrodistillation and headspace solid-phase microextraction (HS-SPME). GC-MS identification results indicated that volatile compounds included terpenes, terpenoids, alcohols, hydrocarbons and ketones [21]. Among them, the content of sesquiterpenoids was 43.2% and 39.4% in the volatile oil obtained by hydrodistillation and HS-SPME, respectively. Compared with our results, we can see that different extraction methods will affect the difference of EOs composition and quality [22]. Also, several researchers have attributed the antibacterial activity of GEOs to these active ingredients (zingiberene, α-farnesene, 6-gingerol, and α-curcumene), arguing that they can affect the permeability and release of intracellular components by attacking cell membranes and cell walls [23,24,25].The determination of DIZ, MIC, and MBC as well as the growth curve of bacteria are used to evaluate the antibacterial activity of GEO. Lei et al. [1] reported that GEO and palygorskite composite exhibited good antibacterial effects against *E. coli* and *S. aureus*. Results of MIC analysis and growth curve from our study indicated the same conclusion. Ju et al. [26] also proved that EOs have broad spectrum antibacterial activity and can effectively resist foodborne pathogenic bacteria. Our results affirm that GEO can significantly inhibit the growth of both the test bacteria, which can be serve as a theoretical basis for effective antimicrobial activity in our study.

In the present study, experimental data showed that *S. aureus* is more susceptible to GEO than *E. coli*. This also confirmed the vulnerability of *S. aureus* compared to *E. coli* which was consistent with the results based on different morphological structure of the two bacteria by Zhang et al. [12]. The surface of *E. coli* has a thick outer lipopolysaccharide layer, while *S. aureus* has a monopeptide layer structure [27]. In this regard, gram-negative cells possess a lipid bilayer, which provides an additional protection against antimicrobial compounds [21].

In the study of antibacterial mechanism of GEOs, we have preliminarily revealed that GEOs directly act on cell membrane, destroy cell membrane structure, and then increase cell membrane permeability, thereby causing the bacteria to lose their basic structural functions and eventually cause the bacterial cell death at certain concentration. Moreover, the hydrophobic compounds of GEO may interact with the lipophilic part of the membrane and isolated mitochondria, disrupting their integrity and function (protein, nucleic acid, energy metabolism, and enzyme activity) [21,28]. Therefore, GEOs might have several ways to affect the microbial cells, leading to their death.

Membrane potential (MP) represents the potential difference between the inside and outside of the bacteria and plays a crucial role in bacterial metabolism. The decrease in MP may be caused by the structural damage to the cell membrane [25,29]. After the GEO addition, the loss of fluorescence indicates that cell membrane depolarization leads to decreased cell metabolic viability and bacterial death [30]. This may be due to the fact that hydrophobic compounds of GEO interact with the lipophilic part of the membrane and isolated mitochondria to destroy its integrity and related function [31].

Proteins are biological macromolecules that exist in the bacterial cell membrane and cytoplasm and perform structural functions in bacteria [32]. The above results also showed that bacterial cell membrane was destroyed by GEO due to the leakage of proteins in the bacteria, especially for some large molecular weight proteins [33]. Previous studies have proved that the hydrophobicity of EOs interferes with the synthesis of bacterial lipid membranes, including some proteins and enzymes on the surface of the membranes, leads to increased permeability of bacterial membranes and leakage of proteins in bacteria, all of which are related to phenolic compounds in EOs [34]. Another study has also shown that cinnamon and clove EOs caused protein leakage in *E. coli* [33]. It was confirmed that GEO treatment caused the leakage of proteins in the bacterial cells by destroying the bacterial cell membrane, leading to a decrease in the protein content of the bacterial cells. In this way, it interferes with the synthesis of certain proteins and enzymes, resulting in the reduction of protein expression in bacterial cells, thus exerting its antibacterial activity [4]. The SDS-PAGE was used only to analyze the intracellular proteins (molecular weight) of two bacteria (*E. coli* and *S. aureus*) after treatment with different concentrations of GEO. Since the migration rate of a protein coated with SDS is inversely proportional to the logarithm of its MW. Therefore, the protein bands in the MIC group and MBC groups were faded, and even some of the protein bands disappeared. The final separation of proteins was almost entirely dependent on the differences in relative MW of polypeptides. It can be concluded that GEO treatment resulted in significant change in the protein amount.

Specifically, according to our SDS-PAGE analysis, we assume that some proteins related to the bacterial energy metabolism, including adenosine triphosphatase (ATPase), alkaline phosphatase (ALPase), and β-galactosidase (β-GAL) might be decreased. Our study speculate that the decrease of these enzymes can be one of the main factors leading to bacterial cell death after GEO treatment [35]. In addition, citrate synthase (CS) and isocitrate dehydrogenase (ICDH) are the key regulator of catalytic reactions in TCA cycle [36]. The decrease of these proteins could have inhibited the TCA cycle and affected respiration. Furthermore, some recognized cell wall stress-stimulating member proteins include penicillin-binding proteins (PBP) and peptidoglycan biosynthesis-related proteins (murB) [37]. The decrease in these proteins indicates that the bacterial membrane was destroyed. Meanwhile, the proteins related to DNA metabolism may suffer a negative impact from GEO treatment. At the same time, it can be postulated that stimulatory reactions of GEO can increase the content of some heat shock proteins (GroE, GrpE, IbpA, and hslO). Their main function is to prevent the protein from denaturation by stimulation and to restore its original biological activity [37].

The integrity of the bacterial cell plasma membrane is a key factor during bacterial growth. Therefore, analyzing the integrity of the cell membrane can further reveal the mechanism of antibacterial action [12]. It has been reported that the indicator for assessing the integrity of cell membranes is the nucleic acid content of bacterial suspension at 260 nm [36]. Involved in DNA replication, transcription, and translation, nucleic acid is an indispensable macromolecular substance in bacteria [37]. Our results indicated that the integrity of the cell membranes of the two bacteria was destroyed under the treatment of GEO, leading to the leakage of nucleic acids from the bacterial cells which may further lead to the failure to carry the genetic information and eventually cause the bacterial death.

Real-time PCR analysis attempted to explain the antibacterial mechanism of GEO at molecular level, and also to further demonstrated the inference of the above experiments. Muthaiyan et al. [37] analyzed the mechanism of EO action on *S. aureus* by transcriptional profiling. They found that orange EO significantly (*P* < 0.05) inhibited the expression of genes encoding cell membrane-associated proteins, as well as some genes involved in enzymes related to DNA metabolism. Another study showed that the treatment of EO can inhibit the activity of β-galactosidase, ATPase and ALP, which can be an important factor for resulting bacterial death [27]. It was reported that oregano EO can inhibit *S. aureus* respiratory metabolism by affecting the metabolites and key enzymes of TCA cycle [4]. Based on the results of transcriptional profiling experiments, we can preliminarily conclude that GEO could inhibit the microbial growth in several ways. Firstly, GEO affected the permeability of the cell membrane resulting in leakage of some macromolecular substances (proteins and nucleic acids) and destruction of energy metabolism. Secondly, GEO could inhibit the respiratory metabolism of bacterial strains by disturbing the TCA cycle. Finally, GEO also could disrupt DNA metabolism by inhibiting DNA replication and DNA repair of key proteins and enzymes.

## 4. Materials and Methods 

### 4.1. Microorganisms and Plant Material

The Gram-positive strain: *Staphylococcus aureus* and Gram-negative strains: *Escherichia coli* were procured from our laboratory depository [38,39]. These two bacteria were revived in the Luria Broth medium (Beijing Land Bridge Technology Co., Ltd., Beijing, China) at 37 °C for 16 h for activation of the test cultures [39]. Tongling white ginger was purchased from Tongling White Ginger Development Co., Ltd. (Tongling, China).

### 4.2. Extraction of Essential Oils

The essential oil was extracted from ginger powder by steam distillation [40] and supercritical CO_2_ extraction [15] according to previous methods, respectively. The extracted essential oil was stored in a refrigerator at 4 °C until the next use.

### 4.3. Chemical Composition Analysis by Gas Chromatography-Mass Spectrometry (GC-MS)

The chemical components of GEO were determined via GC-MS (GC-MS 7890 system, Agilent, Palo Alto, CA, USA). The mixture was separated using a DB-5MS column (30 m × 0.25 mm × 0.25 μm). The analysis conditions were as followed: helium was used as a carrier gas, initial temperature of 50 °C for 2 min, heating to 260 °C at a rate of 5 °C/min, and holding for 10 min. The injector temperature was adjusted at 250 °C. The mass range of 42–350 *m*/*z* was used for mass spectra at 70 eV with the electron source temperature of 280 °C. Components were classified on the basis of mass spectral fragmentation, retention time comparison with authentic constituents, mass spectral, and retention time (NIST 14 Mass Spectral and Wiley Registry™ of Mass Spectral Data) [41,42].

### 4.4. Antibacterial Activity 

#### 4.4.1. Agar Diffusion Assay

The procedure for the evaluation of antibacterial activity of GEO was adopted from previously reported agar diffusion method [39] with a few modifications. Briefly, 20 mL tryptose soya agar (TSA) medium (Beijing Land Bridge Technology Co., Ltd., Beijing, China) was added into each Petri dish. After solidification, 100 µL of each bacterial suspension was uniformly coated on Petri plates. Subsequently, sterile filter paper (6.0 × 1.0 mm) covered with 10 µL GEO (16 mg/mL) was added on the surface of inoculated agar followed by incubation at 37 °C for 24 h. Finally, the diameter of inhibition zone (DIZ) was measured to express the antibacterial activity of GEO and dimethyl sulfoxide (DMSO) was used as control.

#### 4.4.2. Determination of Minimum Inhibitory Concentration (MIC) and Minimum Bactericide Concentration (MBC)

MIC is defined as the lowest concentration of EO with no visible bacterial growth. Whereas, MBC indicated the lowest EO concentration which could stop the complete growth on agar plates [43]. Initially, the stock solution of GEO was suspended in DMSO and transferred to 10 mL sterile Lysogeny broth (LB) medium to obtain the concentration of 16 mg/mL. Afterward, the double dilution method was used to attain the final concentration range from 0.25 to 16 mg/mL. To end with, 50 µL microbial suspensions (1 × 10^7^ cfu/mL) were added followed by incubation at 37 °C for 24 h. MBC was calculated by taking out 100 µL from each tube without visual bacteria growth and then sub culturing on agar plates followed by incubation at 37 °C for 24 h.

#### 4.4.3. Bacterial Growth Curves

The antibacterial activity was reflected by the determination of the bacterial growth curve according to the procedure given by Shu et al. [44]. with a few modifications. The two strains (10^7^ cfu/mL, 0.1 mL) were inoculated into 100 mL sterile LB medium and GEO was added to the bacterial suspension to the 1/2 MIC and MIC concentrations, respectively. DMSO was used as control. Then after, the cultured broths were incubated at 37 °C for 24 h and shaken at 180 rpm in rotary shaker (ZHWY-200B, Jiangsu Shenglan Instrument Manufacturing CO., LTD, Changzhou, China). The turbidity of the culture was monitored by measuring the OD_600nm_ for every 2 h intervals.

### 4.5. Antibacterial Mechanism 

#### 4.5.1. Effect of GEO on the Membrane Potential (MP)

The bacterial cell membrane potential was determined to indicate the effect of GEO on bacterial metabolic activity based on the previously reported method [12] with minor modification. In brief, the bacterial solution was cultured at 24 °C for 12 h and the diluted bacterial suspension (c = 1 × 10^7^ cfu/mL) was treated with different concentrations of GEO (MIC and MBC). DMSO was used instead of GEO as negative control and suspension without GEO was used as a control. After continued culturing for 4 h, the bacteria suspension was centrifuged at 4000 rpm for 10 min to obtain the bacterial pellets, which were washed twice with phosphate buffer saline (PBS, 0.1 M, pH 7.2). After washing, 2 µg/mL Rhodamine 123 dye solution (Rhodamine 123 powder was prepared with methanol to form a stock solution of 1.0 mg/mL, and then diluted to 2 µg/mL with PBS) was added to the pellets and the resulting mixture was stored without direct exposure to light for 30 min. All the mixtures were added to quartz colorimetric dish and fluorescence intensity was determined using Fluorescence spectrophotometer (excitation wavelength of 490 nm and the emission wavelength of 530 nm) (F97Pro, Shanghai Precision instrument Co., Ltd. Shanghai, China).

#### 4.5.2. SDS-PAGE Electrophoresis of the Bacterial Cell Proteins

Bacterial protein loss after 4 h treatment with different concentrations of GEO was evaluated by sodium dodecyl sulfate-polyacrylamide gel electrophoresis (SDS-PAGE) based on the method of Huang et al. [45] and Wang et al. [46]. A bacterial suspension (50 mL) was incubated in a shaker at 37 °C for 12 h, and then centrifuged at 3000 *g* to obtain the cell pellet followed by mixing the pellet with sterile 0.9% saline. GEO with different concentrations (MIC and MBC) were added into bacterial suspension, respectively. And the samples without GEO were treated as control. After 4 h, the pellets obtained by 6000 *g* centrifugation for 10 min were washed twice with PBS (pH = 7.2). The ultrasonic cell disintegrator (power 300 W, interval 1.1 s, 5 min; Ningbo Scientz Biotechnology Co., Ltd., Ningbo, China) was used to break the bacterial cell wall and let it release the proteins. Then after, 25 µL loading buffer were mixed with 100 μL of above bacterial suspension in tubes which were further positioned in boiling water bath for next 30 min. The Coomassie brilliant blue R-250 was used to stain the gel and the separated protein bands were obtained after being decolorized.

#### 4.5.3. Determination of Bacterial Cell Proteins Concentration

After 4 h of GEO treatment, the bacterial suspension was centrifuged at 3000 *g* for 10 min to obtain the cell pellet which was washed twice until the OD_600nm_ value of cell suspension reached 2.0 by using PBS (pH = 7.2). Then after, the obtained bacterial cells were further broken in an ultrasonic cell disintegrator (power 300 W, interval 1.1 s, 5 min). The bacterial suspension was centrifuged at 6000 *g* for 10 min and obtained cell pellet was placed on ice. Finally, the protein concentration of all the samples was determined by BCA protein kit (Beijing Labgic Technology Co., Ltd., Beijing, China).

#### 4.5.4. Effect of GEO on Cell Membrane Integrity

The destruction of cell membrane integrity is expressed by determination of the nucleic acid content in the bacterial suspension. The specific measurement method was used based on previous reports [47] with minor modifications. The bacterial suspensions of the two bacteria were centrifuged at 3000 *g* for 10 min and pellets were washed twice using PBS (pH = 7.2). And then, re-centrifugation at 3000 *g* was carried out for 5 min to obtain the pellet. Sterile 0.9% saline was added to replace the LB medium, and different concentrations of GEO (MIC and MBC) were added to the experimental group and control group was without GEO. After continued cultivation in rotary shaker at 37 °C, 2 mL of the bacterial suspension was removed at every 4 h intervals. The supernatant was obtained by centrifugation at 6000 rpm for 5 min, and OD_260nm_ was measured by UV-vis spectrophotometer (UV-2100, Unico Instrument Co., Ltd., Shanghai, China) to indicate the nucleic acid content in the bacterial suspension.

#### 4.5.5. RNA Extraction and Real-Time PCR

The total RNA extraction was carried out following the descriptions given by Muthaiyan et al. [37] and Zhang et al. [36] with slight modifications. Based on the MIC determination results, 1/2 MIC concentration and MIC concentration of GEO were added to the log-phase bacteria and then treated bacteria were cultured for 30 min at 37 °C. The control group without GEO was also incubated for 30 min. Subsequently, all the bacterial samples were centrifuged at 10,000× *g* for 3 min to collect the bacterial pellets followed by washing with PBS. According to the manufacturer’s instructions, total RNA was extracted using 1 mL RNA Isolater Total RNA Extraction reagent (Nanjing Vazyme Biotech Co., Ltd., Nanjing, China.). The purity of RNA was determined by A_260_/A_280_. Reverse transcription was performed using a Vazyme reverse transcription kit. The cDNA was preserved at −80 °C. The LightCycler 480 SYBR Green I Master kit (Shanghai Bestway Enterprise Co., Ltd., Shanghai, China.) was used for quantitative fluorescence PCR detection. RT-PCR was performed using ABI PRISM 7300 Sequence Detection (Applied Biosystems, Carlsbad, CA, USA). The expression of bacterial lysis related genes (Table S1) was analyzed by using the 2^−ΔΔCt^ method. 16S rRNA gene was used as an internal control gene. Each sample was tested with at least three independent readings to ensure the data reproducibility.

### 4.6. Statistical Analysis

All the experiments were conducted in triplicates, and the experimental data were analyzed using IBM SPSS Statistics 22.0 software (SPSS Inc., Armonk, NY, USA). The significance level (*P* < 0.05) was expressed by single-factor analysis of variance (ANOVA). For intuitive analysis of the data, the Origin Pro 8.0 software was used (Origin Lab, Northampton, MA, USA).

## 5. Conclusions

Based on our findings, zingiberene and α-curcumene were identified as the main chemical constituents of GEOs obtained by two different methods. At the same time, the GEOs possessed excellent antibacterial activity against two foodborne microorganisms, with *S. aureus* being more sensitive to GEOs than *E. coli*. Our study concluded that the antibacterial mechanism of GEOs can be described as damage to the bacterial cell membrane which leads to leakage of macromolecular substances such as bacterial proteins and nucleic acids and ultimately results in a decline of bacterial metabolic activity and eventually bacterial cell death. The GEO treatment could affect the physiological activity of test organisms via affecting the expression of some genes encoding key enzymes related to cell lysis, further elucidating the antibacterial mechanism at the molecular level. Our study encourages the value added application of GEOs which represent a promising approach as novel natural and efficient antibacterial agents to supplant chemically synthesized preservatives in the future. Moreover, GEOs contain a variety of bioactive compounds which may indicate the involvement of other related mechanisms for the resulting antibacterial action. Therefore, further research should be conducted to fully elucidate the interactions between the various mechanisms involved and better explain the rationality of GEO as an effective food preservative with a wider scope of action.

## Figures and Tables

**Table 1:** Analysis of volatile components of GEO extracted by two extraction methods.

No	RT (min)	RI	Compounds	Content (Peak area%)	
				Supercritical CO_2_ Extraction	Steam Distillation
1	5.84	710	α-Pinene	1.934	nd
2 3	6.273 6.417	723 727	Camphene Sabinene	3.012 2.651	nd nd
4	7.039	745	α-Phellandrene	2.287	nd
5	8.405	785	β-Phellandrene	3.699	2.567
6	10.426	828	Borneol	0.648	0.279
7 8	13.246 16.473	880 932	Farnesene β-Bisabolene	1.145 2.643	1.135 2.876
9	18.952	969	Elemene	1.203	1.018
10	19.295	974	α-Selinene	0.901	1.836
11	20.612	993	γ-Elemene	0.721	1.709
12	21.314	1003	α-Curcumene	10.221	12.043
13	21.706	1009	Zingiberene	37.549	35.651
14	21.975	1013	α-Farnesene	3.12	4.52
15	22.396	1018	Zingiberone	6.591	9.023
16	22.98	1027	Elemol	0.902	1.021
17	33.542	1178	Geraniol	1.542	1.382
18	36.943	1216	Geranialdehyde	2.031	2.463
19	48.345	1544	6-Gingerol	1.026	0.120
20	48.445	1548	Octadecadienoic acid	0.901	0.682
Total				84.727	78.325

Note: The “nd” indicates not detected; RT, retention time; RI, retention index determined using the n-alkanes C7-C30 on DB-5MS column (30 m × 0.25 mm × 0.25 μm) in the GC-MS analysis.

**Table 2:** Determination of DIZ, MIC and MBC in GEO against *E. coli* and *S. aureus.*

Bacterial Species	DIZ (mm)	MIC (mg/mL)	MBC (mg/mL)
*E. coil*	12.3 ± 0.6 ^b^	2.0	4.0
*S. aureus*	17.1 ± 0.8 ^a^	1.0	2.0

DIZ, diameter of inhibition zone, the values were represented as the means of three replicates ± standard deviation (SD); MIC, minimum inhibitory concentration; MBC, minimum bactericide concentration. The different lowercase letters (e.g., ^a^, ^b^) represents significant difference at *P* < 0.05.

**Table 3:** Effects of GEO on generation time of *E. coli* and *S. aureus.*

Time	*E. coli*			*S. aureus*		
	Control	MIC	MBC	Control	MIC	MBC
2 h	0.97 ± 0.05 ^a^	0.75 ± 0.04 ^b^	0.61 ± 0.02 ^c^	1.17 ± 0.21 ^A^	0.72 ± 0.004 ^B^	0.60 ± 0.08 ^C^
4 h	3.38 ± 0.17 ^a^	1.97 ± 0.08 ^b^	1.24 ± 0.04 ^c^	3.59 ± 0.23 ^A^	2.14 ± 0.12 ^B^	1.21 ± 0.1 ^C^
6 h	7.32 ± 0.29 ^a^	5.39 ± 0.21 ^b^	1.86 ± 0.05 ^c^	8.25 ± 0.34 ^A^	5.04 ± 0.32 ^B^	1.84 ± 0.23 ^C^
8 h	13.32 ± 0.56 ^a^	9.98 ± 0.42 ^b^	2.51 ± 0.06 ^c^	15.82 ± 0.86 ^A^	9.84 ± 0.45 ^B^	2.45 ± 0.37 ^C^
10 h	24.11 ± 0.91 ^a^	17.14 ± 0.68 ^b^	3.15 ± 0.08 ^c^	27.80 ± 1.45 ^A^	15.51 ± 1.43 ^B^	3.08 ± 0.28 ^C^
12 h	40.38 ± 1.04 ^a^	26.77 ± 0.89 ^b^	3.79 ± 0.09 ^c^	50.97 ± 1.87 ^A^	24.57 ± 1.67 ^B^	3.72 ± 0.39 ^C^
14 h	68.17 ± 2.01 ^a^	36.09 ± 1.01 ^b^	4.43 ± 0.12 ^c^	83.50 ± 2.05 ^A^	34.02 ± 1.83 ^B^	4.36 ± 0.69 ^C^
16 h	104.8 ± 4.27 ^a^	42.31 ± 1.23 ^b^	5.08 ± 0.12 ^c^	152.58 ± 5.65 ^A^	47.97 ± 2.57 ^B^	4.99 ± 0.65 ^C^
18 h	129.16 ± 5.03 ^a^	47.65 ± 1.78 ^b^	5.69 ± 0.13 ^c^	266.26 ± 7.01 ^A^	57.42 ± 3.65 ^B^	5.67 ± 0.59 ^C^
20 h	143.66 ± 6.21 ^a^	52.99 ± 1.83 ^b^	6.38 ± 0.17 ^c^	364.16 ± 8.98 ^A^	66.68 ± 3.98 ^B^	6.338 ± 0.76 ^C^
22 h	158.36 ± 6.82 ^a^	58.33 ± 2.03 ^b^	7.05 ± 0.16 ^c^	402.89 ± 9.94 ^A^	73.37 ± 4.97 ^B^	7.04 ± 0.38 ^C^
24 h	175.07 ± 7.34 ^a^	65.09 ± 3.01 ^b^	7.69 ± 0.21 ^c^	441.47 ± 10.13 ^A^	80.05 ± 5.05 ^B^	7.75 ± 0.98 ^C^

The different lowercase letters (e.g., ^a^, ^b^, ^c^) represents significant difference at *P* < 0.05 among three groups of *E. coli*; The different capital letters (e.g., ^A^, ^B^, ^C^) represents significant difference at *P* < 0.05 among three groups of *S. aureus*.

**Table 4:** Effects of GEO on the specific growth rate of *E. coli* and *S. aureus.*

Time	*E. coli *			*S. aureus *		
	Control	MIC	MBC	Control	MIC	MBC
2 h	0.71 ± 0.02 ^a^	0.92 ± 0.03 ^b^	1.13 ± 0.06 ^c^	0.59 ± 0.02 ^A^	0.97 ± 0.031 ^B^	1.14 ± 0.13 ^C^
4 h	0.20 ± 0.01 ^a^	0.35 ± 0.02 ^b^	0.56 ± 0.02 ^c^	0.19 ± 0.04 ^A^	0.32 ± 0.024 ^B^	0.56 ± 0.09 ^C^
6 h	0.09 ± 0.005 ^a^	0.13 ± 0.03 ^b^	0.37 ± 0.01 ^c^	0.08 ± 0.03 ^A^	0.137 ± 0.004 ^B^	0.37 ± 0.06 ^C^
8 h	0.05 ± 0.002 ^a^	0.07 ± 0.003 ^a^	0.28 ± 0.04 ^b^	0.043 ± 0.003 ^A^	0.07 ± 0.007 ^A^	0.28 ± 0.05 ^B^
10 h	0.03 ± 0.003 ^a^	0.04 ± 0.012 ^a^	0.22 ± 0.03 ^b^	0.025 ± 0.006 ^A^	0.044 ± 0.0056 ^A^	0.22 ± 0.01 ^B^
12 h	0.02 ± 0.001 ^a^	0.026 ± 0.01 ^a^	0.18 ± 0.04 ^b^	0.014 ± 0.0006 ^A^	0.0282 ± 0.004 ^B^	0.186 ± 0.014 ^C^
14 h	0.01 ± 0.0005 ^a^	0.019 ± 0.01 ^a^	0.16 ± 0.01 ^b^	0.008 ± 0.0001 ^A^	0.029 ± 0.0054 ^B^	0.158 ± 0.031 ^C^
16 h	0.007 ± 0.001 ^a^	0.016 ± 0.01 ^b^	0.14 ± 0.01 ^c^	0.0045 ± 0.0001 ^A^	0.0146 ± 0.004 ^B^	0.138 ± 0.021 ^C^
18 h	0.005 ± 0.001 ^a^	0.015 ± 0.01 ^b^	0.12 ± 0.003 ^c^	0.0026 ± 0.0003 ^A^	0.012 ± 0.0001 ^B^	0.12 ± 0.014 ^C^
20 h	0.005 ± 0.002 ^a^	0.013 ± 0.01 ^b^	0.11 ± 0.001 ^c^	0.0019 ± 0.0001 ^A^	0.01 ± 0.0002 ^B^	0.1 ± 0.005 ^C^
22 h	0.004 ± 0.002 ^a^	0.012 ± 0.01 ^b^	0.10 ± 0.005 ^c^	0.0017 ± 0.0002 ^A^	0.009 ± 0.0003 ^B^	0.098 ± 0.004 ^C^
24 h	0.0039 ± 0.003 ^a^	0.011 ± 0.003 ^b^	0.09 ± 0.001 ^c^	0.0015 ± 0.0003 ^A^	0.008 ± 0.0005 ^B^	0.089 ± 0.003 ^C^

The different lowercase letters (e.g., ^a^, ^b^, ^c^) represents significant difference at *P* < 0.05 among three groups of *E. coli*; The different capital letters (e.g., ^A^, ^B^, ^C^) represents significant difference at *P* < 0.05 among three groups of *S. aureus*.

## Supplementary Materials

The following are available online, Table S1: List of primers used in the present study.
