# Supplementary material for: Antibacterial Activity and Mechanism of Ginger Essential Oil against Escherichia coli and Staphylococcus aureus
Source: Molecules. 2020 Aug 30;25(17):3955. doi: 10.3390/molecules25173955 (PMC7504760; doi:10.3390/molecules25173955)
Supplement: Supplementary file 1 [file molecules-25-03955-s001.pdf]

Supplement Table 1 List of primers used in the present study

Genes	Primer	Sequence
E. c ATPase	forward	TACTCCGGGATGACTGACGT
	reverse	AGTAACGTGCGCCAAACAAC
E. c ALPase	forward	CATGCTGCGAATCCTTGTGG
	reverse	CCAGCGTGTTACCCTCCTTT
E. c β-GAL	forward	ATACTGTCGTCGTCCCCTCA
	reverse	AACAACCCGTCGGATTCTCC
E. c CS	forward	CCATTTGTTTACCCGCGCAA
	reverse	GCGTTCTGTTCATGGTCAGC
E. c ICDH	forward	GTACTGCGCCGAAATATGCC
	reverse	CCATCCATCAGACGCTCGAA
E. c OGDH	forward	CGACGTTCCAGCAGTTACCT
	reverse	TGCGTTAATGAGCTGCAGGA
E. c DLST	forward	AAGAGAAAGCGTCCACTCCG
	reverse	TGGCGTCGAGATTGTGTTCA
E. c DLD	forward	TATGAAACCGCCACCTTCCC
	reverse	GATTGCCAGGCCGATTTCAC
E. c 16S rRNA	forward	CGTCAAGTCATCATGGCCCT
	reverse	TCACCGTGGCATTCTGATCC
E. c PBP	forward	GGCAGGCAGAGGAAACGATA
	reverse	CATCATAGGTCGGTGCGGAA
E. c murB	forward	TGCCAGCTAAAAGGGATGCA
	reverse	CAGTTGCACCACATCTTCGC
E. c clpA	forward	CCGTAACCGTCTCGACAACA
	reverse	CTGATCCAGCTGAACCTGCA
E. c GroEL	forward	CGCCAAGAAGATCCAGGTGT
	reverse	ATCTGCGCCTTGATCTGCTT
E. c IbpA	forward	GAGTAATGGCGGCTACCCTC
	reverse	CACCTTTCACCACCAGCAGA
E. c holA	forward	CACTGCGTGCGTTGTTTGAT
	reverse	TCCGTTCGTGTCAGGAGTTG
E. c RecF	forward	GCAGATTGGTCGCGTCATTC
	reverse	TCGCCCTGTTTGTCTTTGGT
S. a 16S rRNA	forward	GCAACGCGAAGAACCTTACC
	reverse	CCCAACATCTCACGACACGA
S. a ATPase	forward	CTGTCCCTGTTGCCAAAACG
	reverse	TCCCCTTGTGCTTCCTCAAC
S. a ALPase	forward	ATGCTGCACACGTGACTTCT
	reverse	TGTTCGTGGCAAGGTCGTAA
S.a β-GAL	forward	ATCCTTGCTTGCCTCGTCTC

	reverse	CTCCGTCACTTGAACCTGCT
S. a CS	forward	TGCACATTTCGATCCTGATGC
	reverse	TGTCTTACTCGAGCAAACGC
S. a ICDH	forward	GGTGATGGAATTGGACCGGA
	reverse	TGGCCAGCTAGCACTTCTTT
S. a OGDH	forward	AACTGCTGGATGGACTGGTG
	reverse	TCTTGTGCTGCTCTCGTACG
S. a DLST	forward	CACCGGCATCAACACAAACA
	reverse	GCTGTTTTCTTTCTGCGTGACA
S. a DLD	forward	ACTGGTGGTGTTGAAGGCTT
	reverse	TTTGTGCGCTCTTTTCGTCC
S. a PBP	forward	AAGCAGCCTAAACGTGGTGA
	reverse	GCATCCATGACAACCGCAAA
S. a murB	forward	GTGAAGGTGGTATTCGCGGT
	reverse	TTCAAGGCCAGTAAGTGCGT
S. a ClpB	forward	GCATTAGAGCGTCGTTTCCA
	reverse	CGATCAGACAATTCAGCGGC
S. a hslO	forward	GCCAGGTGCCAAAGATGAAA
	reverse	AGCCTCGCCCAATCCTTTAA
S. a GrpE	forward	ATAGAACGTGCACTTCAAATTGA
	reverse	CGCCAGATTCAAAATCAGGGT
S. a holA	forward	TGGAGATGTGCCTGAATTGG
	reverse	TGGTGCAATCTCTGTTTCGT
S. a RecN	forward	GCGTCAGGTGGAGAACTTTC
	reverse	GCAACTTGTGGCAAGTGAGA
